# Effects of cholesterol on the anionic magnetite nanoparticle-induced deformation and poration of giant lipid vesicles†

**DOI:** 10.1039/d2ra03199j

**Published:** 2022-10-04

**Authors:** Salma Akter, Mohammad Abu Sayem Karal, Sharif Hasan, Md. Kabir Ahamed, Marzuk Ahmed, Shareef Ahammed

**Affiliations:** Department of Physics, Bangladesh University of Engineering and Technology Dhaka 1000 Bangladesh asayem221@phy.buet.ac.bd +880-2-58613046 +880-2-9665613; Radiation, Transport and Waste Safety Division, Bangladesh Atomic Energy Regulatory Authority Agargaon Dhaka 1207 Bangladesh

## Abstract

We have investigated the effects of cholesterol on the deformation and poration of giant unilamellar vesicles (GUVs) induced by anionic magnetite nanoparticles (NPs). Negatively charged lipid, neutral lipid, and cholesterol were used to prepare the charged GUVs (surface charge density of membranes – 0.16 C m^−2^), while only neutral lipid and cholesterol were used to prepare the neutral GUVs. Cholesterol content varied from 0 to 40 mole% for preparing the biologically relevant membranes. The degree of deformation has been characterized by compactness, the value of which remains at 1.0 for spherical GUVs. The value of compactness increases with time for both membranes, but this increase depends on cholesterol content. The average compactness decreases with cholesterol content, and at 60 min, the values are 1.280 ± 0.002 and 1.131 ± 0.010 for 0 and 40 mole% cholesterol containing charged GUVs. The average compactness is relatively lower for neutral GUVs for the corresponding cholesterol. Membrane poration has been investigated by the leakage of calcein, which indicates a two-state transition model. The fraction of deformation is higher for charged GUVs than for neutral ones, while the fraction of poration shows the opposite result. Both the fractions decrease with cholesterol content.

## Abstract 2

Cholesterol inhibits the anionic magnetite nanoparticles-induced deformation of charged and neutral giant lipid vesicles and lipid membrane poration of these vesicles.

## Graphical abstract

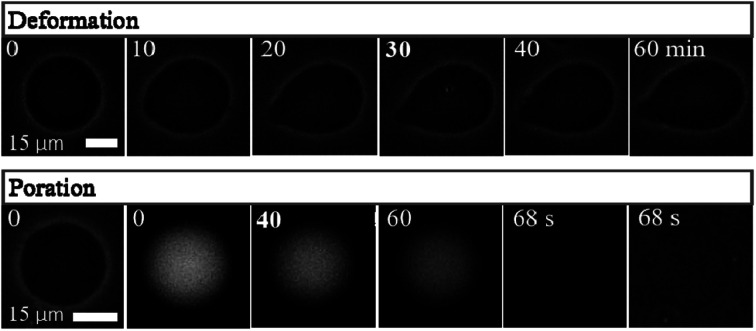

## 1. Introduction

Nanoparticles (NPs) are fascinating objects because of their possible applications in certain diseases, including cancer.^1,2^ Based on NPs, a new method for delivering drugs to targeted regions of the human body is being considered.^3^ In addition, several reports have confirmed the antibacterial and anticarcinogenic effects of NPs.^4–8^ In contrast, negative effects of NPs such as environmental pollution,^9–13^ and cardiovascular and pulmonary diseases^14,15^ have also been reported. The emission of NPs into the environment from various sources is one of the key reasons for the substantial number of deaths and extent of illness in cardiorespiratory diseases.^16,17^ Biomedical implants, contrast agents in MRI, insecticides, and food product processing are some of the common sources of NPs entering the human body.^18–20^ Magnetite NPs can be derived from burning fuel in the iron industry, printer toners, stoves, *etc.* The abundance of magnetite NPs was identified in the human brain; these NPs are prolific in urban areas. Such magnetite NPs can enter the brain *via* the olfactory nerve and can cause build-up of reactive oxygen species in cells. Enhanced reactive oxygen species production links to neurodegenerative diseases (*e.g.*, Alzheimer's).^21^ Magnetite NPs were also found in amyloid plaques (aggregates of misfolded proteins).^22^ Such proteins are responsible for causing Alzheimer's disease. Hence, the exploration of the impact of magnetite NPs in cells/vesicles is important for understanding the possible changes in membranes due to NPs.

Cell membranes, whose major constituents are lipids and cholesterol, are the main boundaries for the interaction of NPs with cells. Among these components, cholesterol (up to 50 mol% of lipid bilayer content) plays a vital role in the operation of real biological and biochemical systems.^23^ Giant unilamellar vesicles (GUVs) of size similar to cells have been used to investigate the interaction of peptides, toxins, and NPs.^24,25^ In addition, GUVs have been used to investigate the poration using mechanical and electrical tension.^26,27^ In several studies, it has been found that cholesterol affects the membrane rigidity, such as bending and elastic modulus.^28–31^ The lipid-specific bending rigidity under various cholesterol has been reported.^32,33^ Addition of cholesterol increases the bending modulus of neutral and charged GUVs.^34–37^

So far, numerous features of lipid vesicles induced by NPs have been considered.^38^ The cationic core–shell magnetic NPs tended to bind with anionic charged membranes.^39^ Various types of shape change, such as protrusion and pearling, in neutral GUVs were observed by encapsulating cationic NPs.^40^ The binding of NPs in membranes causes GUV deformation and poration.^39,41^ Recently, the interaction of anionic magnetite NPs (same NPs as used here) with GUVs on the deformation and poration of cholesterol free charged and neutral GUVs has been investigated.^42^ It has been considered that for DOPC lipid the terminus P^−^ of the dipole (P^−^–N^+^) is tightly attached to the lipid's main molecular structure, while the terminus N^+^ has relative freedom. The electric field is due to the interaction of the terminus N^+^ with the anionic NPs, raising the (P^−^–N^+^) vector's tilt angle. Hence, the normal component of the dipole with respect to the membrane surface increases with the adsorption of anionic NPs.^25,42^ As biological (*i.e.*, human) cells contain cholesterol in their membranes, it is indispensable to know the influence of cholesterol on the lipid vesicle deformation and membrane pore formation induced by anionic NPs. In these investigations, we have considered the interaction of anionic magnetite NPs with biologically-relevant charged and neutral GUVs containing various concentrations of cholesterol in their membranes. Under physiological conditions, the cholesterol mole fraction in these membrane systems ranges from 0 to 40 mole%. More precisely, we have studied the influence of cholesterol on the vesicle deformation and membrane poration induced by anionic magnetite NPs.

## 2. Materials and methods

### 2.1 Chemicals and reagents

Negatively charged lipid 1,2-dioleoyl-*sn*-glycero-3-phospho-(1′-*rac*-glycerol) (sodium salt) (DOPG) and neutral lipid 1,2-dioleoyl-*sn*-glycero-3-phosphocholine (DOPC) were bought from Avanti Polar Lipids Inc. (Alabaster, AL). Cholesterol (chol) was bought from WAKO pharmaceuticals (Japan). O, O′-Bis (2-aminoethyl) ethyleneglycol- *N*,*N*,*N*′,*N*′,-tetraacetic acid (EGTA), piperazine-1, 4-bis (2-ethanesulfonic acid) (PIPES) and bovine serum albumin (BSA) were bought from Sigma-Aldrich (Germany). Ferric chloride anhydrous (FeCl_3_) and ferrous chloride tetra hydrate (FeCl_2_·4H_2_O) were bought from Merck (Germany).

### 2.2 Green synthesis of magnetite NPs

The NPs were prepared *via* the green synthesis method, and the particle size was 18 nm with zeta potential −21.3 mV.^42,43^ The detailed characterization of this NPs is described in our previous paper.^43^ A brief description of the synthesis of the NPs is provided. At first, 60 g paste of *Ipomoea aquatica* leaves was mixed into 0.40 L water (deionized) and kept at 80 °C for 4 hours at 800 rpm. 5 mM FeCl_2_·4H_2_O of amount 20 mL and 10 mM FeCl_3_ of same amount were mixed together by keeping the temperature at 60 °C under 800 rpm and 10 min later, an amount of 5 mL leaf extracts was added into the mixture. After another 10 min, 100 mL 10 mM NaOH was poured into the solution of the mixture. During NaOH addition, the NPs were formed in the solution. The biological molecules of leaf extracts acted as both stabilizers and reducing agents, while FeCl_3_ and FeCl_2_·4H_2_O acted as precursors for preparing the NPs. The description to prepare the various concentrations of NPs is provided in the ESI 1†.

### 2.3 Synthesis of GUVs

The DOPG/DOPC/chol (70/30/0) (70/30/0 specifies molar ratio), DOPG/DOPC/chol (46/39/15), DOPG/DOPC/chol (43/28/29), and DOPG/DOPC/chol (40/20/40)-GUVs were prepared in a physiological buffer (10 mM PIPES, 150 mM NaCl, pH 7.0, 1 mM EGTA) using the natural swelling method.^44^ The surface charge density of these charged membranes was almost the same (−0.16 C m^−2^) for 0, 15, 29 and 40% chol.^37^ For the preparation of charged GUVs, at first, 1 mM DOPG, DOPC and cholesterol (total amount of the mixture was 200 μL) were taken into a glass vial (4.5 mL), which was slightly shaked and kept without any motion for 1 minute to get a homogeneous composition mixture. The solution was dried with a mild flow of nitrogen gas to produce a thin and homogeneous lipid film. Then, the glass vial was kept in a vacuum desiccator for 12 hours. After this, the sample was pre-hydrated for 8 min at 45 °C by pouring 20 μL MilliQ water into the vial, followed by incubation for 3.5 hours at 37 °C with 1 mL 0.10 M sucrose in buffer. To prepare the fluorescent probe (calcein) encapsulated GUVs, we used 1 mM calcein with 0.10 M sucrose in buffer for incubation of the suspension of vesicles. To prepare the neutral membranes containing cholesterol, DOPC/chol (100/0), DOPC/chol (85/15), DOPC/chol (71/29), DOPC/chol (60/40)-GUVs were prepared in 0.10 M sucrose containing MilliQ water. The membrane filtration was used to purify the GUVs.^45,46^ 200 μL purified suspension of GUVs (internal solution 0.10 M sucrose and external solution 0.10 M) was taken into a hand-made microchamber. 100 μL different NPs concentrations were added into the 200 μL GUVs suspension in the microchamber. The microchamber was basically a U-shaped silicone rubber spacer, which was inserted onto a glass slide. The NPs mixed uniformly in the vesicle's suspension. The effective NPs concentrations in the suspension of GUVs were 2.00, 3.33 and 4.70 μg mL^−1^. The 0.10% (w/v) BSA coating was given to the slide glass along with the microchamber to reduce the strong attraction of the membranes to glass. A phase contrast fluorescent microscope (Olympus IX-73, Japan) with 20× objective was used for observing the GUVs by keeping the stage temperature 25 ± 1 °C. The charge-coupled device (CCD) camera (Olympus DP22, Japan) was used to record the images.

### 2.4 Compactness and surface area of a GUV

Compactness (*C*_om_) quantifies the shape change of a GUV, which is defined as follows:^47^1
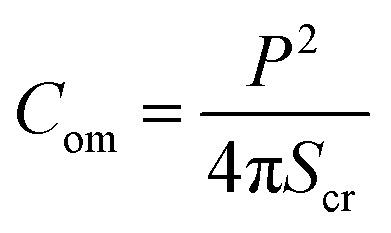
where *P* is the perimeter and *S*_cr_ is the image cross section area of GUVs. The minimal value of *C*_om_ = 1.0 for a circle while it increases with any deviation. MATLAB image processing toolbox was used to calculate the *C*_om_ for each deformed image. The key effect of NPs on GUVs is the increase of total surface area of GUVs (*S*_GUV_). So, we connect *S*_GUV_ with the change of *C*_om_. The surface area of a deformed GUV is defined as follows,2
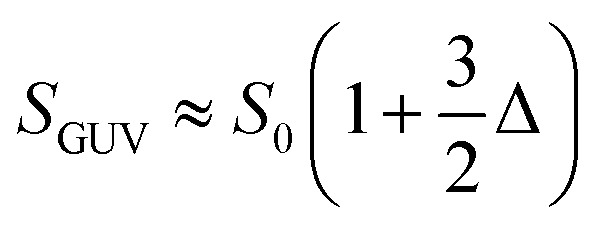
where, *S*_0_ is the surface area of a circular GUVs, 
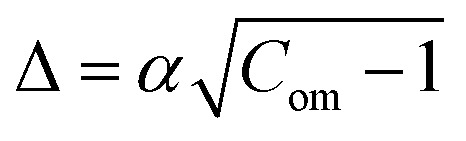
 and 
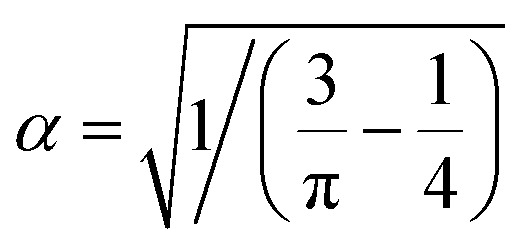
. Hence, the following relation is obtained,3
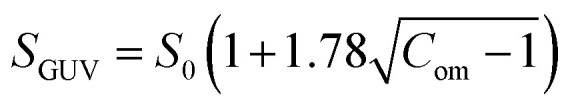


### 2.5 Fraction of deformation and poration of GUVs

The fraction of deformed GUVs (*Fr*_d_) and the fraction of pore formed GUVs (*Fr*_p_) were calculated by measuring the probability of deformation and poration among all the measured GUVs. We explain here how to calculate these fractions. At a concentration of NPs, 100 μL NPs were interacted with 200 μL purified GUV's suspension. The total volume of the NPs-GUVs suspension in the microchamber was 300 μL. During interaction, the images of vesicles were taken at different times, such as 0, 10, 20, 30, 40, 50 and 60 min, by keeping the focus at a fixed position. Then, similar experiments were done for other chambers. The number of deformed GUVs and pore formed GUVs were calculated among all the examined GUVs from several images in each time. We calculated the *Fr*_d_ and *Fr*_p_ at different times for an independent experiment. The same procedure was performed for several independent experiments and the values of *Fr*_d_ and *Fr*_p_ for each independent experiment were obtained. The average value with standard deviation of *Fr*_d_ and *Fr*_p_ was calculated at each defined time and for each cholesterol content.

### 2.6 NPs-induced leakage of calcein from the inside of GUVs

To investigate the NPs-induced poration of GUVs, 100 μL NPs were added to calcein encapsulated GUV's suspension of amount 200 μL. We focused on one GUV during interaction. The GUV was recorded using a CCD camera connected with a fluorescence microscopic. The starting time of poration in the membranes occurred when the leakage of calcein decreased rapidly. The time dependent leakage of calcein for several ‘single GUVs’ was observed for various concentrations of NPs and for various content of cholesterol. The average time of poration was calculated for various cholesterol containing charged and neutral membranes.

## 3. Results

### 3.1 Deformation and compactness of charged DOPG/DOPC/chol and neutral DOPC/chol-GUVs in the presence of 3.33 μg mL^−1^ NPs

**Fig. 1:**
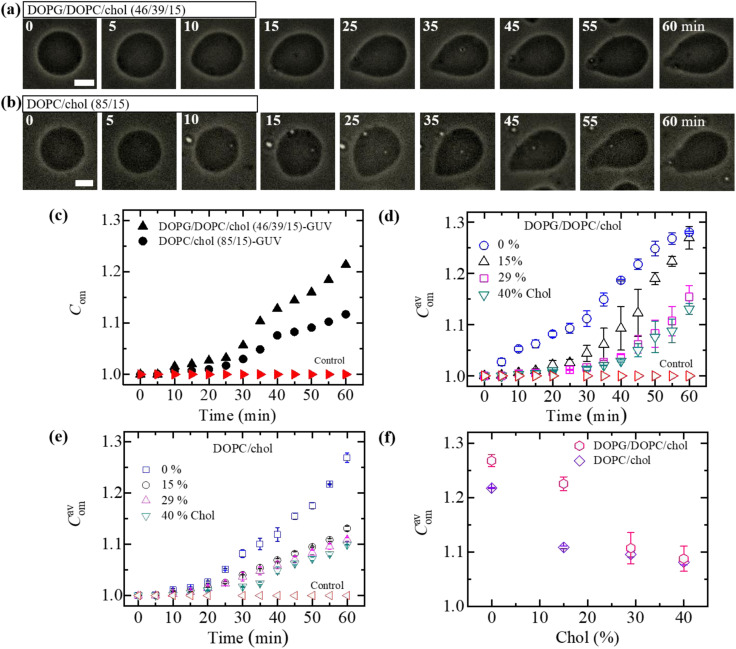
The deformation and compactness (*C*_om_) of charged DOPG/DOPC/chol-GUVs and neutral DOPC/chol-GUVs induced by 3.33 μg mL^−1^ NPs. Phase contrast microscopic images indicate the deformation of a (a) charged GUV and (b) neutral GUV containing 15% chol. The number on each image indicates the time in minute after interacting of NPs. The scale bar is 15 μm. (c) The time dependent *C*_om_ of GUVs as presented in (a and b). The time dependent average compactness (*C*^av^_om_) of (d) charged GUVs and (e) neutral GUVs containing 0, 15, 29 and 40% chol. (f) The decrement of *C*^av^_om_ with cholesterol at 55 min. The data obtained from several independent experiments shows the average value with standard deviation.

At first, we investigated the effects of cholesterol (chol) on the NPs-induced deformation of DOPG/DOPC/chol-GUVs (*i.e.*, charged GUVs). Fig. 1(a) shows the effects of the interaction of 3.33 μg mL^−1^ NPs with a ‘single DOPG/DOPC/chol (46/39/15)-GUV’. In the absence of NPs, the GUV exhibits a spherical structure at 0 min. This form does not alter within the first 9 min following the addition of NPs. A small deformation is initiated at 10 min, while a large deformation is visible from 45 to 60 minutes. The similar deformation is also observed for the other GUVs which is presented in Section ESI 2.† This deformation is very similar to that observed in the cholesterol free membranes.^42^ The degree of deformation is characterized by its compactness, *C*_om_. At 0 min, *C*_om_ is 1.0. The values of *C*_om_ for GUV (Fig. 1(a)) are 1.000, 1.015, 1.020, 1.032, 1.103, 1.144, 1.184 and 1.214 at 5, 10, 15, 25, 35, 45, 55 and 60 min, respectively. The time course of *C*_om_ is presented in Fig. 1(c). Fig. 1(d) shows the change in average compactness, *C*^av^_om_ for 0, 15, 29 and 40 mole% chol in the charged GUVs. The values of *C*^av^_om_ at 60 min are 1.280 ± 0.002, 1.269 ± 0.022, 1.154 ± 0.022 and 1.131 ± 0.010 for 0, 15, 29 and 40% chol in charged membranes, respectively. Next, the effects of cholesterol on the NPs-induced deformation of DOPC/chol-GUVs (*i.e.*, neutral GUVs) have been investigated. The phase contrast images of a ‘single DOPC/chol (85/15)-GUV’ due to the interaction of 3.33 μg mL^−1^ NPs are presented in Fig. 1(b). Before the addition of NPs into the vicinity of GUV, it is spherical in shape at 0 min. The shape deviated from the spherical geometry with time, resulting in the value of *C*_om_ increases. For the first 14 min, the GUV remains intact and shows increased deformation from 15 to 60 min. At 0 min, *C*_om_ is 1.0. The values of *C*_om_ are 1.001, 1.006, 1.007, 1.017, 1.049, 1,083, 1.103 and 1.117 at 5, 10, 15, 25, 35, 45, 55 and 60 min, respectively. The time course of *C*_om_ is also shown in Fig. 1(c). The time dependent *C*^av^_om_ is shown in Fig. 1(e) for various cholesterol containing neutral membranes.

The values of *C*^av^_om_ decrease with the increase of cholesterol for both neutral and charged GUVs (Fig. 1(f)). This investigation suggests that cholesterol inhibits deformation of both types of vesicles in the presence of NPs. The value of *C*^av^_om_ is higher for charged vesicles compared to neutral ones (Fig. 1(f)). The deformation was not observed in the control experiments. In addition, the solutions containing the NPs were matched osmotically to the GUV solution.

### 3.2 Fraction of deformed charged DOPG/DOPC/chol-GUVs in the presence of 3.33 μg mL^−1^ NPs

**Fig. 2:**
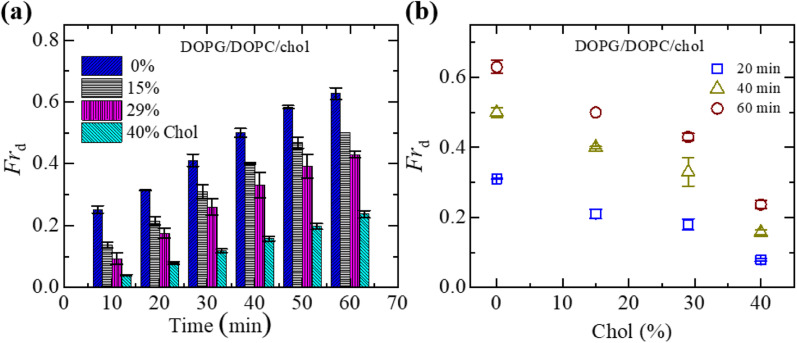
Fraction of deformed (*Fr*_d_) DOPG/DOPC/chol-GUVs in the presence of 3.33 μg mL^−1^ NPs. (a) Bar graph of *Fr*_d_ with time. (b) The linear decrement of *Fr*_d_ with cholesterol at 20, 40 and 60 minutes. The data obtained from several independent experiments shows the average value with standard deviation.

In Section 3.1, the interaction of NPs with a ‘single GUV’ is presented. Here, the interaction of 3.33 μg mL^−1^ NPs with an ensemble of DOPG/DOPC/chol-GUVs is presented. After adding NPs into the GUV's suspension, both the deformed GUVs and intact ones were observed. At first, we have added the NPs in the suspension of charged GUVs containing 15% chol. The images of GUVs were captured at 0, 10, 20, 30, 40, 50 and 60 min in a fixed focusing position. The second and third chamber were subjected to similar experiments. The number of deformed GUVs was measured among all the examined GUVs (number of examined GUVs, *N* = 40–50) from some images at each time. As an example, at 20 min, if 40 single GUVs are counted from several images; out of which 10 GUVs are found to be deformed, the fraction of deformed GUVs (*Fr*_d_) is 0.25 at that time. We have calculated the *Fr*_d_ for different times. These investigations are treated as an independent experiment. The same procedure has been done for 3 independent experiments. The time dependent *Fr*_d_ along with average *Fr*_d_ is described in Section ESI 3.†Fig. 2(a) shows the bar graph of the time dependent average *Fr*_d_ for various cholesterol containing charged GUVs in the presence of 3.33 μg mL^−1^ NPs. The cholesterol dependent Fr_d_ at different times is shown in Fig. 2(b). At 60 min, the values of *Fr*_d_ are 0.63 ± 0.02 for 0% chol, 0.50 ± 0.00 for 15% chol, 0.43 ± 0.01 for 29% chol and 0.24 ± 0.01 for 40% chol. As the cholesterol increases in the charged membranes, the *Fr*_d_ decreases almost linearly. Hence, cholesterol impedes the fraction of deformed GUVs.

### 3.3 Fraction of deformed charged DOPG/DOPC/chol-GUVs in the presence of various NPs

**Fig. 3:**
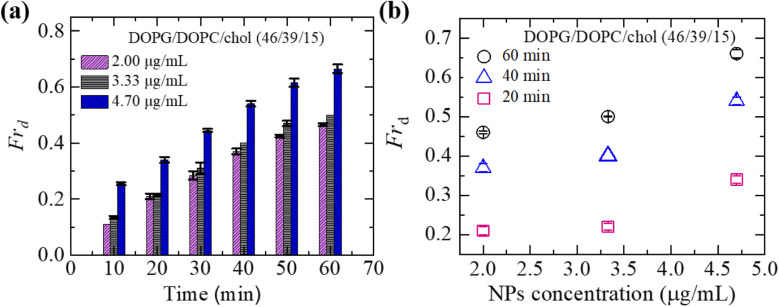
Fraction of deformed (*Fr*_d_) charged GUVs containing 15% chol in the presence of various NPs concentrations. (a) Bar graph of *Fr*_d_ with time at 2.00, 3.33 and 4.7 μg mL^−1^ NPs. (b) The NPs concentration-dependent *Fr*_d_ at 20, 40 and 60 minutes. The data obtained from several independent experiments shows the average value with standard deviation.

To understand the interaction trend of NPs more clearly, we have used various concentrations of NPs. The time-dependent bar graph of *Fr*_d_ of charged GUVs containing 15% chol at 2.00, 3.33 and 4.70 μg mL^−1^ NPs concentrations is shown in Fig. 3(a). The increasing trend of *Fr*_d_ with time is observed for all NPs. The NPs concentration-dependent *Fr*_d_ for different times is shown in Fig. 3(b). The value of *Fr*_d_ at 50 min is 0.43 ± 0.01 for 2.00 μg mL^−1^ while 0.47 ± 0.01 for 3.33 μg mL^−1^ and 0.62 ± 0.02 for 4.70 μg mL^−1^ NPs. Hence, the value of *Fr*_d_ depends on the NPs concentration interacted with vesicles.

### 3.4 Leakage of encapsulating calcein of charged DOPG/DOPC/chol-GUVs induced by 3.33 μg mL^−1^ NPs

**Fig. 4:**
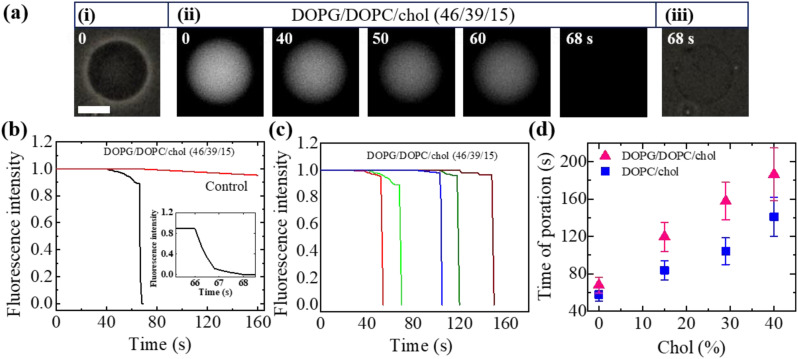
The leakage of calcein from the inside of DOPG/DOPC/chol-GUVs induced by NPs of 3.33 μg mL^−1^. (a) The fluorescence images in (ii) show the change of encapsulating calcein of charged GUV containing 15% chol. The number on each image is the time in second after adding NPs in the GUV's suspension. The scale bar is 15 μm. (b) The time dependent normalized fluorescence intensity of GUV as presented in (a). The rapid change of fluorescence intensity with time is shown in inset. (c) Under identical condition of (b), the change of normalized fluorescence intensity for 5 different charged GUV containing 15% chol. (d) The cholesterol dependent average time of poration in charged and neutral GUVs. The data obtained from several independent experiments shows the average value with standard deviation.

Here, we describe the results obtained from the leakage of calcein from the inside of DOPG/DOPC/chol-GUVs. The leakage experiment for DOPC/chol-GUVs is presented in Section ESI 4.†Fig. 4(a) depicts a representative experimental result of the interaction of 3.33 μg mL^−1^ NPs with a charged GUV containing 15% chol. At 0 s (*i.e.*, in the absence of NPs) of Fig. 4(a)(i), the GUV exhibits a great contrast in the microscopic image (phase contrast) due to the difference in refractive indices between the internal sucrose and external glucose solution of GUV. In Fig. 4(a)(ii), the same GUV is shown in a fluorescence microscopic image, where high contrast of calcein (white color) is observed inside of vesicle. After adding NPs solution in the suspension of vesicles, the inside fluorescence intensity of GUV remains unchanged until 40 s, followed by a rapid decrease in fluorescence intensity as shown in Fig. 4(a)(ii). From 40 to 65 s, the intensity decreases by a negligible amount, and at 66 s, intensity rapidly reduces to zero. The fluorescence intensity becomes zero within 1 s (see inset of Fig. 4(b)). After finishing leakage of calcein, the same GUV is shown in the phase contrast image at 68 s (Fig. 4(a)(iii)). It indicates that GUV remains intact with undetectable break after complete leakage of encapsulating calcein of GUVs. The sharp decrease in fluorescence intensity is similar to that observed in the peptide-induced leakage of such types fluorescent probes,^24,48^ where the time of sharp decrease indicates the poration in membranes due to NPs. The time course of normalized fluorescence intensity of calcein is shown in Fig. 4(b). As long as the fluorescence intensity in the inside of GUV remains 1.0 (or about to 1.0), no leakage of calcein occurs. In contrast, a rapid decrease of normalized fluorescence intensity indicates pore formation in the membranes of GUVs. The similar investigation was performed for many GUVs and a similar decrease of fluorescence intensity was observed. For an example, we present 5 different charged GUVs containing 15% chol in Fig. 4(c) under 3.33 μg mL^−1^ NPs. Fig. 4(c) depicts the stochastic nature of pore formation. It means poration in membranes occurs at different times although the NPs concentration is the same for several similar sized charged GUVs containing 15% chol. We have calculated the average time of poration for different cholesterol containing membranes. The average time of pore formation increases with cholesterol for charged and neutral membranes as shown in Fig. 4(d). Hence, pore formation in vesicles is hindered due to the increase of cholesterol content in the membranes. The average time of poration for neutral membranes is lower than that of the charged ones (Fig. 4(d)).

### 3.5 Fraction of deformed and pore formed charged DOPG/DOPC/chol and neutral DOPC/chol-GUVs

**Fig. 5:**
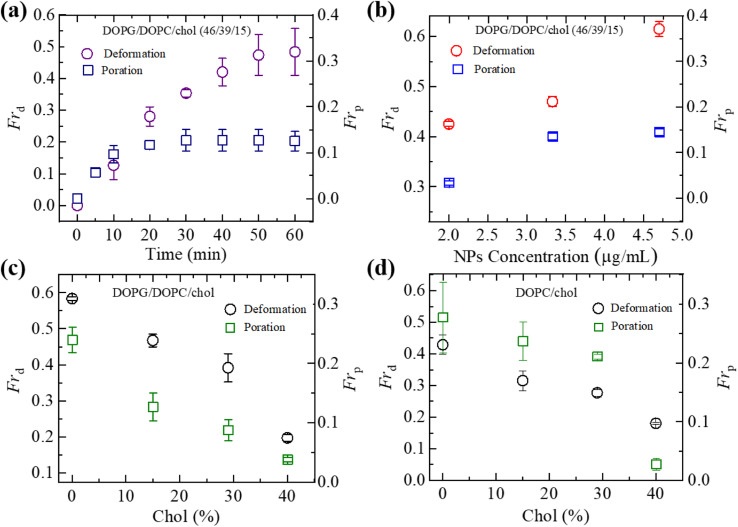
Fraction of deformation (*Fr*_d_) and fraction of poration (*Fr*_p_) of charged DOPG/DOPC/chol and neutral DOPC/chol-GUVs. (a) The time dependent *Fr*_d_ and *Fr*_p_ of charged GUVs containing 15% chol under 3.33 μg mL^−1^ NPs. (b) The NPs concentration dependent *Fr*_d_ and *Fr*_p_ of charged GUVs containing 15% chol at 50 min. (c) The cholesterol dependent *Fr*_d_ and *Fr*_p_ of charged GUVs at 50 min under 3.33 μg mL^−1^ NPs. (d) The cholesterol dependent *Fr*_d_ and *Fr*_p_ of neutral GUVs at 50 min under 3.33 μg mL^−1^ NPs. The data obtained from several independent experiments shows the average value with standard deviation.

We have computed the fraction of deformed GUVs (*Fr*_d_) and the fraction of pore formed GUVs (*Fr*_p_) for several cholesterol containing charged and neutral GUVs to determine the likelihood of deformation and pore formation. Fig. 5(a) shows such fractions with time for charged GUVs containing 15% chol in the presence of 3.33 μg mL^−1^ NPs. The value of *Fr*_d_ increases until 50 min and then levels off with time. On the other hand, *Fr*_p_ increases until 30 min and then remains constant. The NPs concentration dependent *Fr*_d_ and *Fr*_p_ of charged GUVs containing 15% chol at 50 min is shown in Fig. 5(b). In both situations, the fractions grow as the concentration of NPs increases. At 2.00 μg mL^−1^, the values of *Fr*_d_ and *Fr*_p_ are 0.43 ± 0.01 and 0.03 ± 0.01, respectively. At 3.33 μg mL^−1^, these values are 0.47 ± 0.01 and 0.13 ± 0.01, respectively. The cholesterol dependent *Fr*_d_ and *Fr*_p_ under 3.33 μg mL^−1^ at 50 min is shown in Fig. 5(c), where both factions decrease as cholesterol increases.

We have further investigated the *Fr*_d_ and *Fr*_p_ for cholesterol containing neutral membranes to compare the results with the charged ones. Fig. 5(d) shows the change of *Fr*_d_ and *Fr*_p_ with time for cholesterol containing neutral GUVs under 3.33 μg mL^−1^ NPs. It is evident that such fractions decrease with increasing cholesterol content in the neutral GUVs. As an example, for DOPC/chol (85/15)-GUVs, *Fr*_d_ and *Fr*_p_ are 0.32 ± 0.03 and 0.24 ± 0.03, respectively, whereas for DOPC/chol (60/40)-GUVs these values are 0.18 ± 0.01 and 0.04 ± 0.01, respectively.

**Fig. 6:**
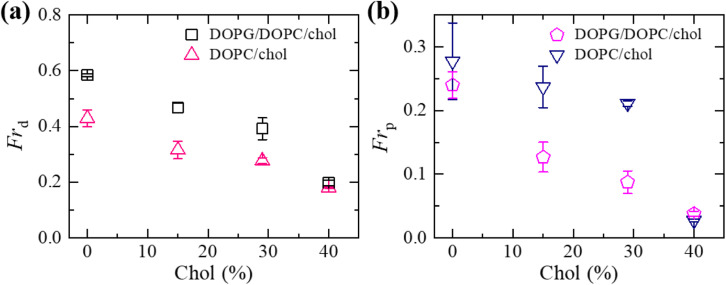
Comparison of the fraction of deformation (*Fr*_d_) and the fraction of poration (*Fr*_p_) between the cholesterol containing charged and neutral GUVs in the presence of 3.33 μg mL^−1^ NPs at 50 min. (a) The cholesterol dependent *Fr*_d_. (b) The cholesterol dependent *Fr*_p_. The data obtained from several independent experiments shows the average value with standard deviation.

We have compared the *Fr*_d_ and *Fr*_p_ between the cholesterol containing charged and neutral GUVs in the presence of 3.33 μg mL^−1^ NPs (Fig. 6). Both fractions show decreasing trend with increasing cholesterol. The values of *Fr*_d_ for different cholesterol containing charged membranes are higher compared to cholesterol containing neutral membranes (Fig. 6(a)). The scenario for *Fr*_p_ shows opposite fashion (Fig. 6(b)). In the case of DOPG/DOPC/chol (43/28/29)-GUVs and DOPC/chol (71/29)-GUVs, the *Fr*_d_ values are 0.39 ± 0.04 and 0.28 ± 0.01, respectively. On the other hand, for DOPG/DOPC/chol (46/39/15)-GUVs and DOPC/chol (85/15)-GUVs, the *Fr*_p_ are 0.13 ± 0.02 and 0.24 ± 0.03, respectively.

## 4. Discussion

The influence of cholesterol on the deformation and poration in DOPG/DOPC/chol and DOPC/chol-GUVs has been investigated. The degree of deformation (*i.e.*, compactness) and the fraction of deformation increase with time, but these values decrease with cholesterol. The values of compactness and fraction of deformation are higher for charged membranes (surface charge density – 0.16 C m^−2^) compared to neutral membranes. Cholesterol inhibits the NPs induced poration in membranes, and the average time of poration in neutral membranes is lower than charged ones. The fraction of poration increases with time and then becomes steady. This fraction for neutral membranes is higher than that for charged ones.

The hydrophilicity of DOPC lipid is determined by the formation of a dipole due to (P^−^–N^+^) group of lipid molecules. The separation between the PO_2_^−^ and N^+^ group in the dipole, is ∼0.5 nm, which is somewhat a large dipole of ∼ 20 Debye.^49^ In general, this dipole vector is oriented at angles in the range of 50−80° with respect to the normal to the membrane surface.^50^ As for the DOPG lipid, OH^−^ groups form several short dipoles contributing to the hydrophilicity, having a bond length of 0.097 nm of OH^−^. Therefore, the dipole moment per DOPG molecule is ∼ 1.76 Debye.^51^ In the dipole of DOPC lipid, terminus P^−^ displaces with the whole molecule as it is firmly attached to the lipid's main structure, wherein terminus N^+^ can move more freely under the effect of external electric field induced by anionic NPs. Negatively charged DOPG molecules create an electric field near the bilayer, and in the bilayer, it repels the anionic NPs away from the membrane surface. Thus, interaction of NPs with the N^+^ terminus causes an increase in the angle of the (P^−^–N^+^) dipole vector tilt. This dipole tilt decreases the area of the outer layer of the bilayer affecting the area mismatch between the two monolayers of membranes.

The ‘bilayer coupling model’^52^ can explain the mechanical state of lipid vesicles in which the shape of a closed bilayer such as GUVs is determined by the elastic energy (*W*_el_) at its minimum condition. This energy is related to the bending energy of membranes (*W*_b_) but not the monolayer elastic stretching. The minimum elastic energy is determined by the area difference between the two layers of a bilayer for a given area and volume of GUV.^53–55^ The shape change (*i.e.*, deformation) of vesicles was well explained by the ADE (area difference elasticity) model.^56,57^ In this model, the monolayer area is not constant to the equilibrium area but stretches elastically to increase the bilayer's nonlocal elastic energy. In this case, the elastic energy is the sum of *W*_b_ and the relative monolayer stretching (*W*_r_) energy as expressed by following relations.4
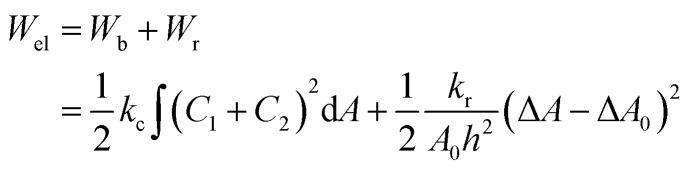
where, *k*_c_ is the local bending modulus and *k*_r_ is the nonlocal bending modulus. *C*_1_, and *C*_2_ are the two principal curvatures. Integration is made over the neutral surface of bilayer with surface area *A*_0_. The difference in area between the external monolayer and the internal monolayer of membranes is Δ*A* = *A*^out^ − *A*^in^ at stretched state. The separation between the neutral surfaces of two layers is *h*. The difference in area between the two layers of bilayer at equilibrium (*i.e.*, unstretched state) is Δ*A*_0_ = *A*^out^_o_ − *A*^in^_o_. In ADE model, the GUV's shape is determined by the *W*_el_ at its minimum condition for a definite area *A* and volume *V*, and also for area difference Δ*A*_0_ at equilibrium (relaxed) state. Under constant volume of GUVs, the deformation of vesicles from spherical shape is due to (Δ*A* − Δ*A*_0_)^2^. Therefore, the degree of deformation (*i.e.*, compactness) of vesicles increases with time for the adsorption of NPs (Fig. 1). This model can also explain various types of deformation as shown in Fig. 1(a) and ESI 2.† The ‘bilayer coupling model’ and the ADE model are two possible mechanisms causing the deformation of GUVs.

We have incorporated cholesterol to the membranes and investigated the deformation and poration of vesicles under NPs concentration. Cholesterol inhibits the deformation of spherical-shaped GUVs along with the formation of pores in their membranes. In continuum mechanics,^58,59^ vesicles can be treated as a ‘thin-walled closed shell’ which has several stable forms^60,61^ and hence, the approach was used for analyzing such vesicles.^62–65^ As the vesicle has several stationary structures with various forms of energy (*E*_i_), it maintains the minimum energy (*E*_sph_) for a spherical-shaped vesicle. The energy of deformed GUVs, *E*_def_ ≫ *E*_sph_. The *Fr*_d_ is expressed as follows:^42^5
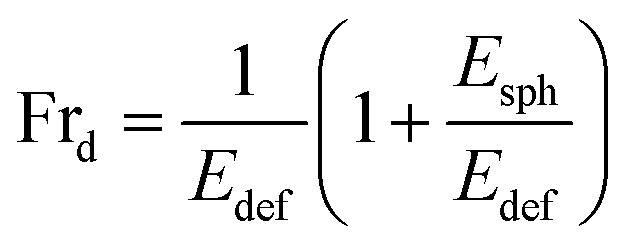


As time progresses, if *E*_sph_ increases due to the area mismatch between the two layers of membranes, the probability of deformed GUVs increases. One of the important mechanical properties, bending modulus of vesicles, is affected by the content of cholesterol in their membranes. The bending modulus is increased in several folds by the increase of cholesterol content in the DOPG/DOPC/chol and also DOPC/chol membranes.^35,37,66^ For that reason, increasing cholesterol concentration in the membranes employ a descending trend of compactness, which supports our results as shown in Fig. 1(f). Incorporation of cholesterol would reduce *E*_sph_, and hence *Fr*_d_ decreases. To transform the spherical-shaped GUVs into other forms, vesicles must overcome the barrier width Δ*E*_bar_ = *E*_bar_ − *E*_sph_, where *E*_bar_ is the energy barrier. The increase of cholesterol increases the energy barrier, and therefore, *Fr*_d_ decreases (Fig. 2(b)). On the other hand, adsorption of more NPs *i.e.*, increase in NPs concentration in the membrane gives rise to the area mismatch between the monolayers and thus affects to increase *Fr*_d_, which supports our study (Fig. 3(b)).

**Fig. 7:**
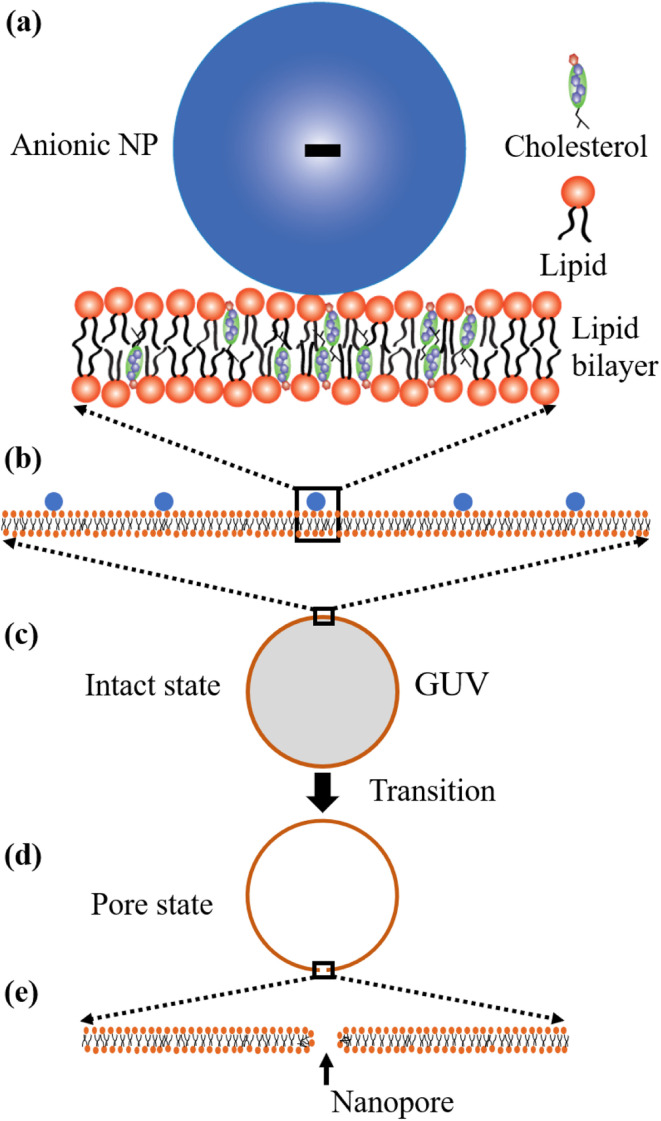
Two-state transition model for NPs induced poration in lipid bilayer. (a–c) Adsorption of NPs in the outer layer of membranes of GUVs. (d and e) Poration in the membranes of GUVs.

Now, we discuss the experimental results on the NPs induced leakage of calcein from GUVs (Fig. 4). Several recent papers have explained the mechanism of antimicrobial peptide-induced leakage of encapsulating probes (*e.g.*, calcein, Alexa fluor).^48,67,68^ The binding of peptide to the external layer of the membranes increases the area of this layer, leading to the stretching of the inner layer of membranes. Such stretching causes positive lateral tension in the internal monolayer of membrane, which induces vesicle poration and, consequently, leakage of calcein. In several experimental observations, pore or rupture formation in GUVs have been investigated due to the stretching of the lipid bilayer.^27,69–71^ At the initial state of poration, the pore radius is too small for the leakage of calcein, and hence a slight change in fluorescent intensity before rapid change at relatively larger sized pores is observed (Fig. 4(b and c) and ESI 4 (b and c)).† The lateral membrane tension induces the penetration of polystyrene NPs across the lipid bilayer of GUVs.^72^ The lateral pressure in membranes induced by adsorbed AuNPs creates membrane rupture or the formation of pores.^73^ Using coarse-grained molecular dynamics simulations, the dynamics along with the mechanism of membrane rupture in the presence of carbon NPs under mechanical stress have been investigated.^74^ The strengthening and weakening effects of large and small NPs on the strength of lipid bilayers have been demonstrated.^74^ In a coarse-grained numerical simulation, Janus NPs diffuse to the stable pore, that sustains after the external stress is removed. As soon as the NPs-lined pore is formed, a slight rise in bilayer tension readily re-opens the pore, permitting transport through the bilayer.^75^ Based on the above discussion, we describe the anionic magnetite NPs induced membrane poration by the two-state transition model^76^ as illustrated in Fig. 7. The first is the ‘intact state’ in which NPs are adsorbed to the membrane's outer monolayer (Fig. 7(a–c)), and the second is the ‘pore state’ in which a pore is formed in the GUV's membrane (Fig. 7(d and e)). As a result, the membrane surface bends in such a way that a toroidal pore is formed in the vesicle membrane. The rate constant (*k*_p_) is the rate of transformation from the intact state to the pore state, which is described by the following well known Arrhenius equation,6
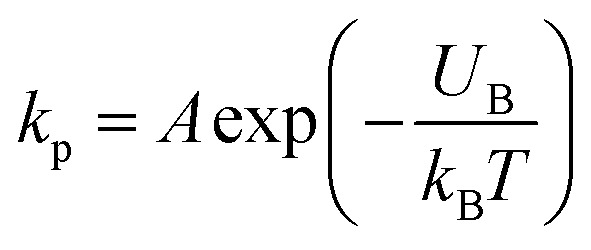
where, *U*_B_ is the barrier energy of a pore, *k*_B_ is the Boltzmann constant, *T* is the absolute temperature and *A* is a constant. However, separate events of this two-state transition happen stochastically. If such transition results in irreversible poration, the fraction of intact state can be defined as the fraction of intact GUVs, *Fr*_intact_ (*t*) at which no leakage of calcein occurs.

In our experiment, this state has been found for both charged and neutral GUVs containing chol at *t* = 0 (Fig. 4 and ESI 4†). This state is sustained for a certain time after interacting of NPs with GUVs, where the fluorescence intensity remains constant. After a certain interval of time, there is a sharp decrease of fluorescence intensity, indicating the pore state. So, the pore state is the state of GUV at which calcein leaks out, and therefore, the fraction of poration, *Fr*_p_ can be expressed as, 1 − *Fr*_intact_(*t*).

Based on these discussion, it can be considered that the adsorption of NPs in the external layer induces stretching such as lateral tension (*σ*_n_) in the bilayer, leading to the creation of transmembrane pore in the GUV's membrane. In the case of a toroidal pore, the free energy of a pre-pore is:^77,78^*U*(*r*,*σ*_n_) = 2π*rΓ* − π*r*^2^(*σ*_n_ + *B*), where *B* is the electrostatic term due to charged lipids, *Γ* is the line tension, and *r* is the radius of a pre-pore. The barrier energy of a prepore, 
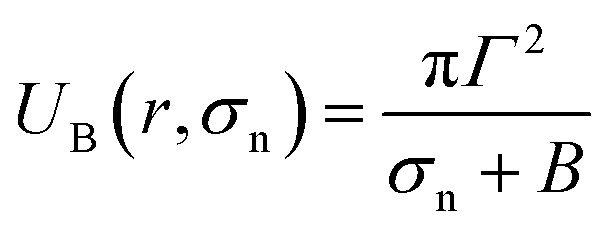
. With the addition of 0 to 40% chol in the membranes of DOPG/DOPC/chol-GUVs (surface charge density – 0.16 C m^−2^), the line tension increased from 12.9 to 14.6 pN.^79^ This increase raises the value of *U*_B_(*r*, *σ*_n_), which decreases the rate of poration (see eqn (6)). With the addition of cholesterol, the time of pore formation increases (Fig. 4(d)). In addition, the fraction of pore-formed GUVs decreases, which supports our findings (Fig. 5(c and d)). It is to be noted that there were few cases where both poration and deformation were occurred in some GUVs in the presence of NPs concentration. In that case, we counted only the poration.

As described previously in this Section, the dipole (P^−^–N^+^) of neutral DOPC/chol-GUVs is attracted more to the anionic NPs than to charged DOPG/DOPC/chol-GUVs, because there is an electrostatic repulsion between OH^−^ and the anionic NPs. In the case of cholesterol containing neutral GUVs, the attractive force between anionic NPs and N^+^ may reach a critical value, resulting in greater fraction of pore formed neutral GUVs than charged ones. On the other hand, in cholesterol containing charged GUVs, the repulsive force between OH^−^ and the anionic NPs may cause less attraction than in neutral GUVs. Hence, area mismatch may increase with a longer time interval in charged GUVs. This may cause a higher fraction of deformed charged GUVs compared to the neutral ones. This discussion is in accordance with the investigation (Fig. 6).

As nanotechnology-based research and industry have grown very rapidly, it is alarming for human beings to protect themselves from the exposure of NPs. From these investigations, it can be hypothesized that adsorption of NPs can change the shape of cells and form pores in their membranes. As the biomembranes contain cholesterol, such cholesterol can decrease the level of exposure in their membranes, but not stop the exposure. The interaction of NPs affects cardiovascular and pulmonary activities^14,15^ resulting in cardiorespiratory diseases^16,17^ with substantial mortality and morbidity. Since NPs-based pesticides and food products are used in agriculture, it may reduce or damage the activity of beneficial bacteria as NPs may also bind their membranes. In contrast, if it is necessary to kill or damage the pathogenic bacteria (bacterial membrane is cholesterol free), these NPs may also be used. To reduce the exposure of NPs, proper safety in nanotechnology-based laboratories and industries is important. In addition, special care should be taken for the drug delivery system using NPs. Therefore, these investigations may help to develop new medical and pharmacological technologies.

## 5. Conclusions

We have investigated the anionic magnetite NPs-induced deformation of spherical-shaped DOPG/DOPC/chol and DOPC/chol-GUVs and also the poration in the membranes of such GUVs. The cholesterol contents are varied from 0 to 40 mole% in both membranes in a physiological buffer. The values of compactness and the fraction of deformed GUVs increase with time for both systems. The deformation started with an insignificant one and then reached to almost a steady value. Both types of deformation decrease with cholesterol content in the membranes. The deformation is explained based on the thin-walled closed shell model, bilayer coupling model, and ADE model. The membrane poration was studied by the leakage of a water-soluble fluorescent probe, calcein, from the inside of GUVs. The two-state transition model can reasonably explain the poration in GUVs. The increased line tension at the edge of a pore is one of the important causes of the inhibition of poration in cholesterol-containing membranes. At a specific cholesterol content, the value of the fraction of deformed vesicle is higher for the charged vesicles as compared to the neutral ones, in which poration shows the opposite trend. Such a trend occurs due to the differences in head's chemical structure of DOPG and DOPC lipids. This research would help to understand the effects of cholesterol on the interaction of anionic NPs with real cells.

## Data availability

The data regarding to support this findings are available upon reasonable request to the corresponding author.

## Author contributions

S. A. and M. A. S. K. designed the work. S. A. and S. H. performed the experiments. S. A., M. K. A. and M. A. analyzed the data. All the authors wrote the paper and discussed thoroughly for improving the manuscript.

## Conflicts of interest

There is no conflict of interest regarding this manuscript.

## Supplementary Material

RA-012-D2RA03199J-s001
