# Supplementary material for: Effects of cholesterol on the anionic magnetite nanoparticle-induced deformation and poration of giant lipid vesicles
Source: RSC Adv. 2022 Oct 4;12(44):28283–94. doi: 10.1039/d2ra03199j (PMC9531538; doi:10.1039/d2ra03199j)
Supplement: RA-012-D2RA03199J-s001 [file RA-012-D2RA03199J-s001.pdf]

Electronic Supplementary Material (ESI) for RSC Advances.
This journal is © The Royal Society of Chemistry 2022

Supplementary Information (SI)

Effects of cholesterol on the anionic magnetite nanoparticles-induced deformation and poration of giant lipid vesicles

Salma Akter[1#], Mohammad Abu Sayem Karal[1#*], Sharif Hasan[1], Md. Kabir Ahamed[1,2], Marzuk Ahmed[1], and Shareef Ahammed[1]

[1]Department of Physics, Bangladesh University of Engineering and Technology, Dhaka-1000, Bangladesh
[2]Radiation, Transport and Waste Safety Division, Bangladesh Atomic Energy Regulatory Authority, Agargaon, Dhaka-1207, Bangladesh

#Authors contributed equally.

*Correspondence should be addressed to:
Email: asayem221@phy.buet.ac.bd

SI 1: Preparation of various concentrations of NPs

Ipomoea aquatica (commonly named as water spinach) was bought from Palashi market in Dhaka, Bangladesh, which is available in the country. A typical *Ipomoea aquatica* leaf is shown in Fig. SI 1(a). The magnetite NPs were prepared using the green synthesis technique,[1,2] which is described in section 2.2 of the main text. The leaf extracts (Fig. SI 1(b)) acted as both stabilizers and reducing agents. At the final stage of the synthesis, the NPs were precipitated at the bottom of glass beaker (Fig. SI 1(c)), which then accumulated using a magnetic bar. The solution in the beaker on the upper side was removed. The synthesized NPs were placed in aluminum foil and drier for 48-72 hours at 60 °C (dried NPs are shown in the inset of Fig. SI 1(c)). The dried NPs were then converted to powder form using a hand mortar. At first, a relatively higher NPs concentration of 0.20 mg/mL was prepared in buffer for charged vesicles and in MilliQ for neutral vesicles, which was then diluted to several concentrations such as 0.006, 0.01, and 0.013 mg/mL. To interact the NPs with lipid vesicles, 100 μL NPs were mixed with 200 μL purified suspension of GUVs in a microchamber. Hence, the total volume of NPs and vesicles suspension was 300 μL. In that case, the corresponding effective concentrations of NPs in the observation microchamber were 2.00, 3.33, and 4.67 μg/mL.

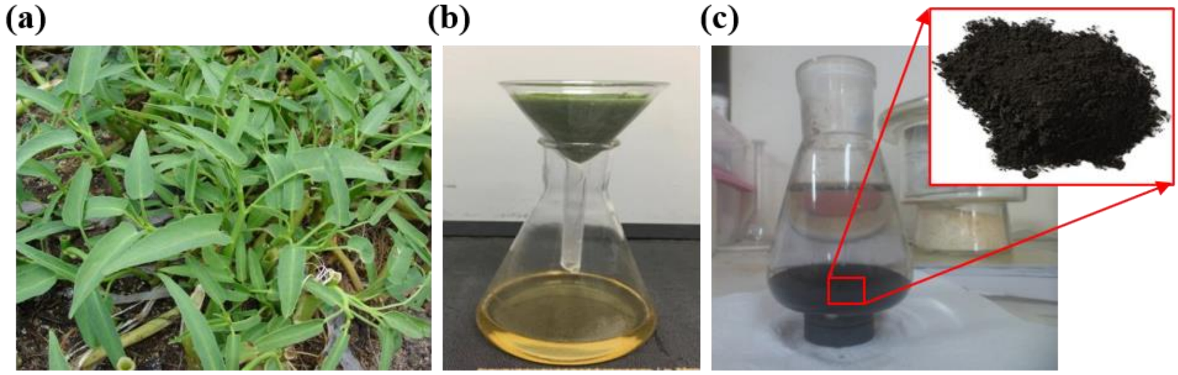

Fig. SI 1. Preparation of magnetite NPs using leaf extracts. (a) *Ipomoea aquatica* leaf. (b) Leaf extracts. (c) NPs in colloidal solution (dried NPs are shown in the inset).

SI 2: Deformation and compactness of DOPG/DOPC/chol-GUVs in the presence of 3.33 μg/mL NPs

In section 3.1 of the main text, it was presented the deformation and the corresponding degree of deformation for a 'single DOPG/DOPC/chol (46/39/15)-GUV' in the presence of 3.33

μg/mL NPs (Fig. 1). Here, we present the deformation of 2nd and 3rd GUVs under the same conditions. In Fig. SI 2a(i, ii), in the absence of NPs, the DOPG/DOPC/chol (46/39/15)-GUVs show a spherical structure at 0 min. A small deformation is visible after 10 min, and a large deformation is visible after 25 to 60 minutes. A similar deformation is seen in the other GUVs. The time course of compactness (C_{om}) of the corresponding GUV as shown in Fig. SI 2(a) is presented in Fig. SI 2(b). At 0 min, C_{om} is 1.0. The values of C_{om} for GUV (i) are obtained 1.000, 1.002, 1.021, 1.041, 1.135, 1.237, 1.300 and 1.334 at 5, 10, 15, 25, 35, 45, 55 and 60 min, respectively, while the values of C_{om} are obtained 1.000, 1.003, 1.010, 1.013, 1.051, 1.117, 1.177 and 1.231 at 5, 15, 20, 25, 35, 45, 55 and 60 min, respectively, in case of GUV (ii).

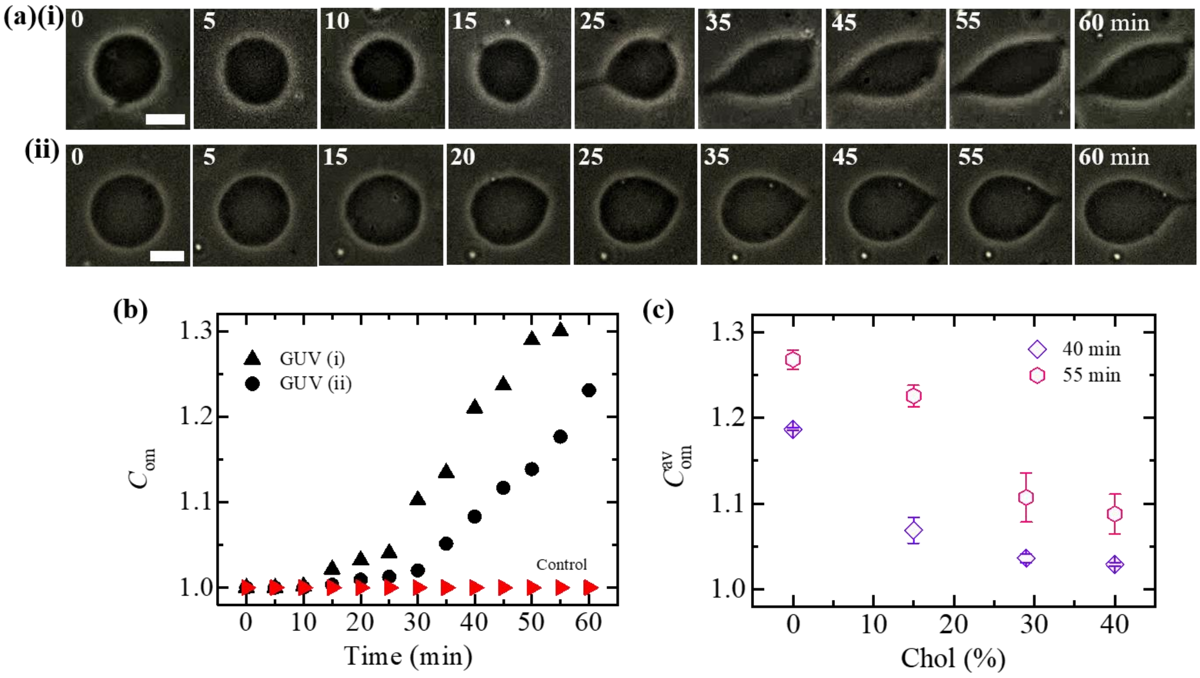

Fig. SI 2. The deformation and compactness (C_{om}) of charged DOPG/DOPC/chol-GUVs induced by NPs of 3.33 μg/mL. (a) Phase contrast images show the deformation of two different DOPG/DOPC/chol (46/39/15)-GUVs. The number on each image indicates the time (minute) after interacting of NPs. The scale bar is 15 μm. (b) The time-dependent C_{om} change of the corresponding GUVs as presented in (a). (c) The average compactness of GUVs at 40 and 50 minutes as a function of cholesterol. The data obtained from several independent experiments shows the average value with standard deviation.

The cholesterol dependent average compactness (C_{om}^{av}) of DOPG/DOPC/chol-GUVs in the presence of 3.33 μg/mL NPs at 40 and 55 minutes is presented in Fig. SI 2(c). These investigations clearly indicate that addition of cholesterol in the membranes of vesicles inhibits the NPs-induced deformation of GUVs.

SI 3: Fraction of deformation of DOPG/DOPC/chol (46/39/15)-GUVs in the presence of 3.33 μg/mL NPs

The deformed GUVs are the GUVs that have deformed after the addition of NPs in the suspension of vesicles. After the interaction of NPs with GUV suspension, both the deformed GUVs and intact ones were observed. In an independent experiment, if 50 GUVs were deformed out of 100 GUVs at 20 min after the addition of NPs, the value of Fr_d = 0.20 at that time. We calculated the Fr_d for different times. The values of Fr_d for DOPG/DOPC/chol (46/39/15)-GUVs at time 0, 10, 20, 30, 40, 50, and 60 min were obtained 0, 0.14, 0.21, 0.31, 0.40, 0.48, and 0.50, respectively (Fig. SI 3(a)). The same procedure has been done for 3 independent experiments. The time dependent average Fr_d at each defined time is shown in Fig. SI 3(b), which increases with time. The average values of Fr_d at 0, 10, 20, 30, 40, 50 and 60 min are obtained 0, 0.15 ± 0.02, 0.22 ± 0.03, 0.29 ± 0.04, 0.40 ± 0.01, 0.48 ± 0.02 and 0.50 ± 0.04, respectively. This data confirmed that Fr_d increased with time.

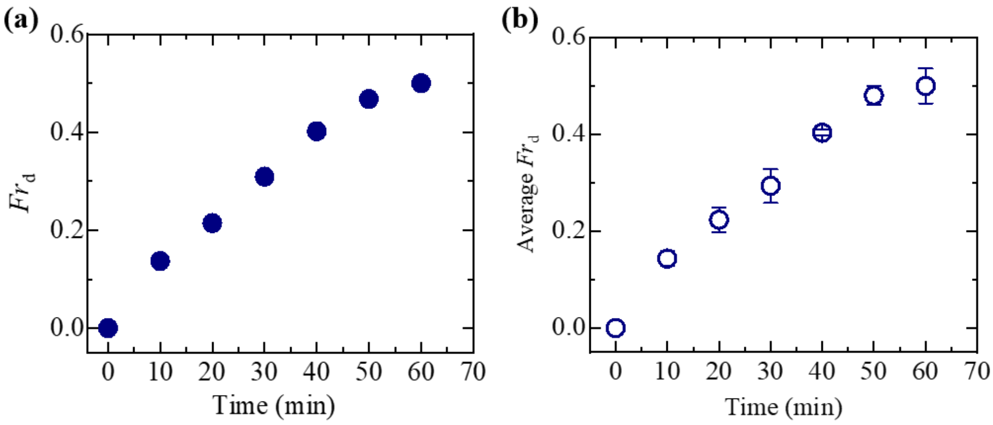

Fig. SI 3. The time course of the fraction of deformed (Fr_d) DOPG/DOPC/chol (46/39/15)-GUVs in the presence of 3.33 μg/mL NPs for (a) one independent experiment and (b) three independent experiments. The data obtained from several independent experiments shows the average value with standard deviation.

SI 4: Leakage of encapsulating calcein of neutral DOPC/chol-GUVs induced by 3.33 μg/mL NPs

In section 3.4 of the main text, we described the leakage of calcein from cholesterol containing charged GUVs. We also investigated, the leakage of calcein from inside of DOPC/chol-GUVs (i.e., neutral GUVs) for checking whether the leakage shows a similar trend like charged vesicles. Fig. SI 4 shows a typical experimental result of the interaction of 3.33 μg/mL NPs with a DOPC/chol (85/15)-GUV. Without the addition of NPs to the vesicle suspension, a spherical shaped GUV is observed in a phase contrast image at 0 s (Fig. SI 4a(i)). In Fig. SI 4a(ii), the same GUV is shown in a fluorescence microscopic image with a high contrast of calcein (white color) in the inside of the GUV. After adding NPs solution to the suspension of GUVs, the fluorescence intensity inside the GUV remained almost unchanged for the first 57 s, while a rapid decrease in intensity occurred at 58 s. The GUV with an undetectable break is shown in the phase contrast image of Fig. SI 4a(iii).

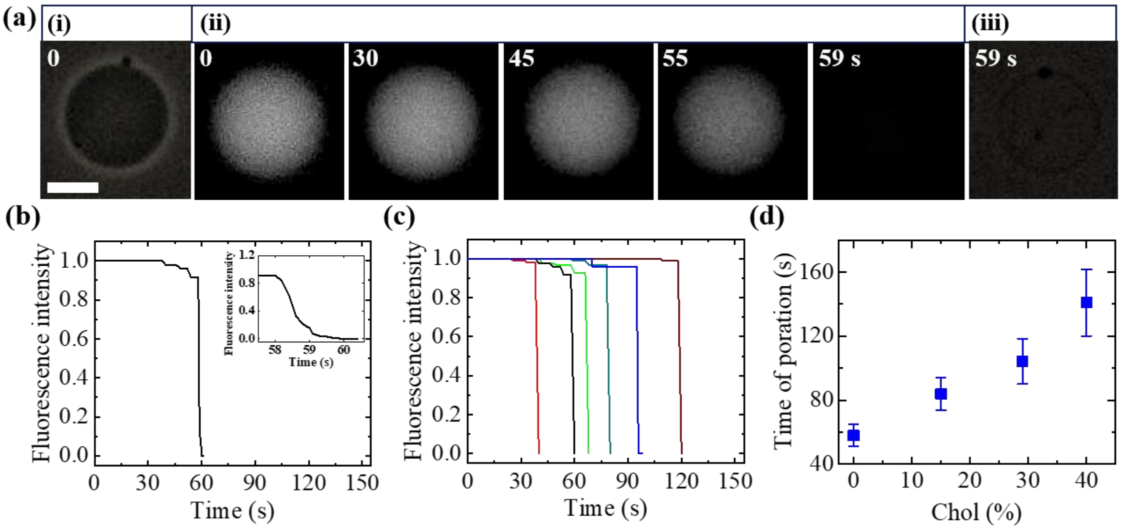

Fig. SI 4. The leakage of calcein from the inside of DOPC/chol-GUVs induced by 3.33 μg/mL NPs. (a) The fluorescence images (ii) show the change of calcein from the inside of DOPC/chol (85/15)-GUV. The number on each image shows the time in seconds after the NPs were added to the suspension of GUVs. The scale bar is 15 μm. (b) The time course of the change in normalized fluorescence intensity of GUV as presented in (a). The rapid change of fluorescence intensity with time is shown in the inset. (c) Under the same condition as (b), the change of normalized fluorescence intensity with time for 6 different DOPC/chol (85/15)-GUV. (d) The average time of poration in neutral GUVs as a function of cholesterol. The data obtained from several independent experiments shows the average value with standard deviation.

Figure SI 4(b) depicts the time course of the change in normalized intensity of the GUV. The fluorescence intensity became zero within 1 s (inset of Fig. SI 4(b)). Figure SI 4(c) depicts the stochastic pore formation of six different DOPC/chol (85/15)-GUVs. A similar type of stochastic pore formation was also observed for other cholesterol containing neutral GUVs. The average time of pore formation for different cholesterol containing membranes is shown in Fig. SI 4(d). The average time increased with cholesterol, which shows a similar trend as observed for charged GUVs (Fig. 4 of the main text). These investigations suggested that cholesterol hindered the NPs-induced pore formation in lipid vesicles.

References

1 M. A. S. Karal, S. Ahammed, V. Levadny, M. Belaya, M. K. Ahamed, M. Ahmed, Z. B. Mahbub and A. K. M. A. Ullah, *Chem. Phys. Lipids*, 2020, 104916.

2 M. M. Zaman, M. A. S. Karal, M. N. I. Khan, A. R. M. Tareq, S. Ahammed, M. Akter, A. Hossain and A. K. M. A. Ullah, *ChemistrySelect*, 2019, **4**, 7824–7831.
